# Neuroprotection by Paeoniflorin against Nuclear Factor Kappa B-Induced Neuroinflammation on Spinal Cord Injury

**DOI:** 10.1155/2018/9865403

**Published:** 2018-12-02

**Authors:** Bin Wang, Wangying Dai, Lijun Shi, Honglin Teng, Xigong Li, Jing Wang, Wujun Geng

**Affiliations:** ^1^Department of Orthopedics, The Second Affiliated Hospital of Zhejiang Chinese Medical University, Hangzhou 310005, China; ^2^Department of Spine Surgery, The First Affiliated Hospital of Wenzhou Medical University, Wenzhou 325035, China; ^3^Department of Pharmacology, The First Affiliated Hospital of Wenzhou Medical University, Wenzhou 325035, China; ^4^Department of Orthopedic Surgery, The First Affiliated Hospital, School of Medicine, Zhejiang University, Hangzhou 310003, China; ^5^Department of Anesthesiology, The First Affiliated Hospital of Wenzhou Medical University, Wenzhou 325035, China

## Abstract

**Background:**

Acute spinal cord injury (SCI) is one of the most common and devastating causes of sensory or motor dysfunction. Nuclear factor-kappa B(NF-*κ*B)-mediated neuroinflammatory responses, in addition to nitric oxide (NO), are key regulatory pathways in SCI. Paeoniflorin (PF), a major active component extracted from Paeonia roots, has been suggested to exert neuroprotective effects in the central nervous system. However, whether PF could improve the motor function after SCI* in vivo* is still unclear.

**Method:**

Immunohistochemical analysis, western blot, real-time quantitative PCR, immunofluorescence staining, and histopathological and behavioral evaluation were used to explore the effects of paeoniflorin after SCI for 14 days.

**Results:**

In this study, PF treatment significantly inhibited NF-*κ*B activation and downregulated the expression of inducible nitric oxide synthase (iNOS), cyclooxygenase-2(COX-2), and Nogo-A. Comparing behavioral and histological changes in SCI and PF treatment groups, we found that PF treatment improved motor function recovery, attenuated the histopathological damage, and increased neuronal survival in the SCI model. PF treatment also reduced expression levels of Bax and c-caspase-3 and increased the expression level of Bcl-2 and cell viabilities. Upregulation of TNF-*α*, IL-6, and IL-1*β* after injury was also prevented by PF.

**Conclusion:**

These results suggest that the neuroprotective effects of PF are related to the inhibition of the NF-*κ*B signaling pathway. And PF may be a therapeutic strategy in spinal cord injury.

## 1. Introduction

Spinal cord injury (SCI) can produce a variety of sensory or motor dysfunction, resulting from several types of acute and chronic central nervous system trauma. SCI pathology is subcategorized into primary and secondary injury. Primary injury is irreversible physical injury to the spine, whereas secondary injury is a series of chemical, reversible secondary pathophysiological changes, including inflammatory reaction, ischemia, apoptosis, and cell and tissue oxidative stress. Neuroinflammatory response is thought to play a pivotal role in secondary injury after SCI [1, 2].

Nuclear factor-kappa B (NF-*κ*B) is a key regulatory transcription factor in immunological inflammatory stress. There are five proteins in the mammalian NF-*κ*B family: p50, p52, RelA/p65, RelB, and c-Rel [3]. RelA, also known as NF-*κ*B p65, is a Rel-associated protein involved in NF-*κ*B heterodimer formation and nuclear translocation and activation [3]. In most normal cells, NF-*κ*B is present as an inactive, I*κ*B-compound conjugate in the cytoplasm [4]. Activation of NF-*κ*B occurs through the degradation of inhibitor kappa B (I-*κ*B) by various signaling mechanisms, causing it to enter the nucleus to combine with DNA and activate gene expression [4]. NF-*κ*B has been shown to transcribe the genes encoding proinflammatory cytokines (TNF-*α*, IL-l*β*, and IL-12), inducible nitric oxide synthase (iNOS), and cyclooxygenase-2 (COX-2) [5]. Previous studies have shown that NF-*κ*B is activated following acute SCI [6] and knockdown of NF-*κ*B in the rat can improve motor function recovery after trauma [7]. Additionally, inhibition of NF-*κ*B-mediated inflammatory responses in SCI rats may improve the hind limb function of rats [8].

After SCI, a series of molecular protein makes up a repulsive environment, which inhibits axons regeneration and nerve function recovery. NO synthase (NOS) is an iron-containing monooxygenase that exists in three isoforms, neuronal NOS (nNOS), endothelial NOS (eNOS), and inducible NOS (iNOS). NOS produces NO* in vivo* and catalyzes the generation of large number of neurotoxic NO molecules, resulting in neuronal cell hypoxia, edema, and apoptosis. The inflammatory stimuli (TNF-*α*, IL-l*β*, and NO derived from iNOS and COX-2) that promote the generation of prostaglandin E2 play a crucial role in the pathogenic processes involved with neurodegenerative diseases and posttraumatic inflammatory reactions after SCI [9, 10]. Since iNOS is a downstream signaling molecule of the NF-*κ*B pathway, iNOS expression may directly affect damage progress. Nogo-A is a well-known axons regeneration inhibitor [11]. The C-terminal of Nogo-A (Nogo-66) binds to Nogo-66 receptor (NgR) transducing the inhibitory signal, unfavorably affecting the cytoskeletal structure of axons and axon growth [12]. Inhibition of Nogo-A expression in rodent and primate model of SCI could promote regeneration of axons and improve functional recovery [13, 14].

Paeoniflorin (PF), a monoterpene glucoside, is the primary active chemical extracted from the roots of Paeonia plants. PF has been reported to have various bioactivities and function in immune regulation, such as the apoptosis induction [15], anti-inflammation [16, 17], immune regulation, pain-relief, and antioxidant activity [18]. Moreover, PF has been reported to have nerve protection effects. In the early 1990s, a Japanese scholar was the first to report that PF can improve learning and memory function decline in an animal model of dementia. Liu et al. found that PF protects microglia by inhibiting the NF-*κ*B and VEGF/Flt-1 signaling pathways, suggesting a potential for use in treating Alzheimer disease (AD) [19]. Several studies have shown that PF has a central neuroprotective effect. However, most previous studies have concentrated on ischemic brain damage, Parkinson's disease, and used other* in vitro *experiments, including the use of PC12 cells [20], glial cells [21], and rat cerebral cortical neurons [22]. However, the effect of PF on motor function improvement after SCI and underlying molecular signaling proteins,* in vivo, *is yet to be reported. Therefore, the aim of this study was to elucidate the effect of PF on neuronal inflammation, oxidative stress, and repair of neurological function, via* in vivo *and* in vitro *studies of NF-*κ*B pathway-mediated inflammatory responses.

## 2. Materials and Methods

### 2.1. Animals

Young adult male and female healthy Sprague–Dawley (SD) rats, weighing 200 to 240 g, were obtained from the Animal Center of Chinese Academy of Sciences, Shanghai, China. At the beginning of the experiment, all animals were fed for 1 week in a standard temperature room (23 ± 0.5°C) and maintained on a 12-h light/dark cycle, with free access to an abundance of water and food. All animal care, breeding, and testing procedures accepted the supervision and inspection of Laboratory Animal Ethics Committee of Wenzhou Medical University and Laboratory Animal Centre of Wenzhou Medical University and fully complied with the Guide for the Care and Use of Laboratory Animals from the National Institutes of Health.

### 2.2. Spinal Cord Injury and PF Administration

Rats were randomly divided into one of the following three groups (n=30 per group): (1) Sham, (2) SCI, and (3) SCI+PF. Rats were anaesthetized with an intraperitoneal injection of pentobarbital sodium (40mg/kg). While fixed on an operating table, skin was prepared and sterilized with povidone iodine. The skin and muscle were incised along the spinous process, and a laminectomy was performed at T9 segmental level vertebrae to form a 2 mm×2 mm open area of the spinal cord. The exposed spinal cord received a crush injury using a vascular clip (15 g force; Oscar, China) for 1 min. Sham rats were not injured and only laminectomy was performed. Hemostasis was used to close the wounds for all groups. Postoperatively, bladders were artificially emptied three times per day.

PF (purity > 98.0%) was purchased from Solarbio (Solarbio Science & Technology, Beijing, China) and dissolved in 0.9 % saline solution. Beginning the day after surgery, Sham and SCI rats were intraperitoneally injected once per day with 0.9% saline, and PF rats were injected with 20mg/kg/day paeoniflorin, until rats were sacrificed.

### 2.3. Motor Function Evaluation

Motor function was evaluated by two trained observers blinded to the experimental procedures, using the Basso-Beattie-Bresnahan (BBB) open field locomotor scale, inclined plane test, and footprint analysis at 1, 3, 7, and 14 days after injury. The range of BBB locomotor scale scores was from 0 (complete paralysis) to 21 (normal locomotion) [23]. The inclined plane test measures the maximum angle of the plane at which a rat could maintain itself for 5 s. Footprint analysis was performed by dipping the animal's hind paws in red dye and allowing it to walk on a walkway with a limited aisle (100 cm×6.5 cm). Footprints were recorded, and blotting paper was compared between three groups [24].

### 2.4. Tissue Preparation

At the designated time points (1, 3, 7, and 14 days) after SCI, rats were deeply reanesthetized before being euthanized. Rats were transcardially perfused with physiological saline solution and 4% paraformaldehyde until the body became fixed. A 2.5-cm section around the lesion epicenter of the spinal cord was removed and postfixed overnight by immersion in the same fixative. Next, the samples were embedded in paraffin and cut into 5-*μ*m thickness transverse and longitudinal sections. Paraffin sections were adhered to SuperFrost plus slides for immunohistochemistry, Hematoxylin–Eosin (HE) and Nissl staining.

### 2.5. Hematoxylin–Eosin (HE) and Nissl Staining

After deparaffinization, sections were hydrated in graded ethanol, followed by HE staining with hematoxylin and eosin at the lesion epicenter. In addition, sections were incubated with Nissl solution (0.1 % Cresyl violet) for 20 min and visualized under a light microscope (BX53, Olympus, Japan) [25]. Neurons containing Nissl bodies were counted per section. Following HE staining, sections were given a pathological score ranging from 0-6, based on the following: 0 = no damage; 1 = 1-5 eosinophilic neurons in gray matter; 2 = 6-10 eosinophilic neurons in gray matter; 3 = more than 10 eosinophilic neurons in gray matter; 4 = less than 1/3 infarction area of gray matter; 5 = 1/3-1/2 infarction area of gray matter; 6 = more than 1/2 infarction area of gray matter.

### 2.6. Immunohistochemistry

Sections from samples obtained on days 1, 3, 7, and 14 after SCI were processed for IHC. Sections were blocked with 3% hydrogen peroxide for 10 min and, after a 15-min rinse in fresh PBS, were boiled (at 98°C) in 0.01 M citrate buffer for 15 min for antigen retrieval, and then blocked with hydrogen peroxide for 60 min. Subsequently, the sections were incubated with the following primary antibodies: iNOS (1:100, Abcam, USA) and COX-2 (1:100, Abcam, USA) overnight at 4°C, followed by horseradish peroxidase-conjugated anti-rat secondary antibodies (1:200, Zhongshan Golden Bridge Biotechnology, Beijing, China). Finally, the immunoreactivity was observed with 0.05% diaminobenzidine (Zhongshan Golden Bridge Biotechnology, Beijing, China), and slides were then restained with hematoxylin, after being dehydrated and transparent, and mounted with 1 drop of neutral resin and cover slipped. Photographs of the ventral horn at a magnification of 400 were acquired using a biological imaging microscope (BX53, Olympus, Tokyo, Japan). Cells positive for iNOS and COX-2 were counted from five randomly selected images per section of the spinal anterior horn and analyzed with Image-Pro Plus. Primary antibodies were replaced by PBS for negative controls.

### 2.7. Western Blot Analysis

Total protein from cells and tissue was extracted by incubation in ice cold RIPA buffer containing 1 mM PMSF and centrifuged at 12,000 rpm for 20 min. Nuclear and cytosolic proteins were extracted using a Nuclear and Cytoplasmic Protein Extraction Kit (Beyotime), in accordance with the manufacturer's instruction. Proteins (30 *μ*g per sample) were separated by 10% sodium dodecyl sulfate (SDS)–polyacrylamide gel electrophoresis and electroblotted onto polyvinylidene difluoride (PVDF) membranes (Millipore, Billerica, MA, USA). Membranes were blocked with 5% fat-free dry milk for 2 h at room temperature and incubated overnight at 4°C with the following primary antibodies: NF-*κ*B p65 (1:1000, Abcam, USA), I*κ*B*α* (1:1000, Abcam, USA), iNOS (1:200, Abcam, USA), COX-2 (1:1000, Abcam, USA), Nogo-A (1:1000, Abcam, USA), Bcl-2 (1:1000, Abcam, USA), and Bax (1:800, Abcam, USA). Subsequently, membranes were incubated with the appropriate horseradish peroxidase-conjugated bovine anti-rat IgG secondary antibody (Beyotime). Protein bands were detected with enhanced chemiluminescent system ECL Plus (Thermo Scientific, USA). GAPDH or Histone 3 was used as a loading control. Band intensity was analyzed with Image J software (Media Cybernetics, Bethesda, MD, USA).

### 2.8. Cell Culture and Viability Assay

PC12 cells were obtained from the American Type Culture Collection (Rockville, MD, USA). Cells were seeded at a density of 2×10^5^ cells/mL in 6-well plates or 2×10^4^ cells/mL in 96-well plates. To induce oxidative stress injury, PC12 cells were incubated with different concentrations of H_2_O_2_ (0, 50, 100, 200, 400 *μ*M) for 2 h. For neuroprotective experiments, PC12 cells were pretreated with PF at various concentrations (0, 50, 100, 200, and 400 *μ*M) for 2 h, followed by exposure to H_2_O_2_ (200 *μ*M) stimulation. Cell viability was evaluated with a Cell Counting Kit-8 (CCK-8, Beyotime) following the manufacturer's instructions.

### 2.9. Real-Time Quantitative PCR

Total RNA was extracted from PC12 cells using TRIzol reagent (ThermoFisher Scientific, Waltham, MA, USA), according the manufacturer's instructions, and was verified by determining the absorbance at 260/280 nm using a spectrophotometer. Reverse transcription was performed using the Revert Aid First Strand cDNA Synthesis Kit (Thermo Scientific, Waltham, MA, USA). A sample of cDNA was used to quantify gene expression by qPCR using a SYBR Green (Bio-Rad, CA, USA) based PCR mixture on an CFX96TM Real-Time system. The amount of mRNA for each gene was normalized by *β*-Actin, and the relative expression levels were calculated using the 2− ΔΔCt method. The sequences of all primers used for the real-time quantitative PCR experiments are presented in Table 1.

### 2.10. Immunofluorescence Staining

Immunofluorescence staining was conducted to analyze NF-*κ*B protein expression inside and outside the nucleus. PC12 cells were fixed with 4% paraformaldehyde for 10 mins, washed 3 times in PBS, and permeabilized with 0.1 % TritonX-100 for 10 min. Subsequently, cells were incubated overnight at 4°C, with the anti-NF-*κ*B antibody. After incubation with the secondary antibody (Zhongshan Golden Bridge Biotechnology, Beijing, China) for 1h at room temperature, nuclei were counter-stained with DAPI. Slides were imaged using a fluorescence microscope (DP70; Olympus, Tokyo, Japan).

### 2.11. Statistical Analysis

Unless indicated, the data are expressed as the mean ± SEM. Statistical evaluation of the data was performed using Student's t test, or one-way analysis of variance (ANOVA) test followed by Dunnett's post hoc test. Statistical significance was accepted for* P* values of < 0.05.

## 3. Results

### 3.1. PF Enhanced Functional Recovery of Hind Limbs in SCI Rats

**Figure 1:**
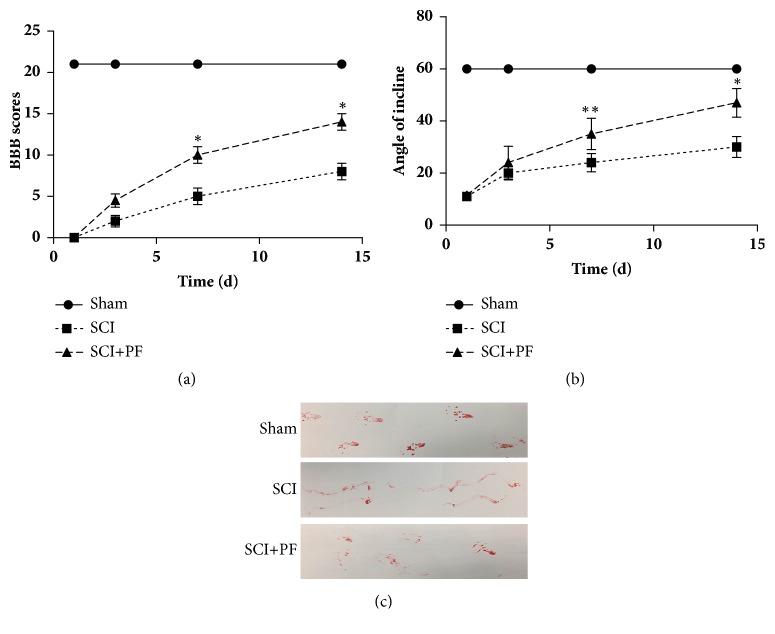
PF enhanced the functional recovery of hind paws on SCI rats. (a) Basso-Beattie-Bresnahan (BBB) open field locomotor scale and (b) inclined plane test. *∗*P<0.01, *∗∗*P<0.05 compared with SCI group on the same day, n=10. (c) Footprint text recorded the hind limbs footsteps of rats at 14 days after SCI.

To determine if PF has a neuroprotective effect, we used the Basso-Beattie-Bresnahan (BBB) open field locomotor scale, inclined plane test, and footprint recordings to analyze the functional recovery of hind paws in SCI rats. SCI immediately induced hind limb paralysis and loss of bladder function, in all rats (SCI and PF groups). Forelimbs are not influenced. Two rats from the SCI group and one rat from the PF group did not survive the injury. Hence, we removed these three rats from all data analysis. As shown in Figures 1(a) and 1(b), hind limb function was significantly recovered in PF rats at 7 and 14 days (P<0.01), compared to SCI rats. No significant difference in BBB scores or angle of incline was observed between SCI and PF rats at 1 or 3 days (P>0.05). At the 14th day after injury, the PF rats were able to exercise joints and move with slight steps. As shown in the footprint recordings (Figure 1(c)), SCI rats had an irregular dragging gait, compared to that of the Sham group that had a normal conformity gait. To sum up, PF intervention enhanced walking function.

### 3.2. PF Reduced Neuronal Trauma and Histopathological Damage in Spinal Cord Tissue

**Figure 2:**
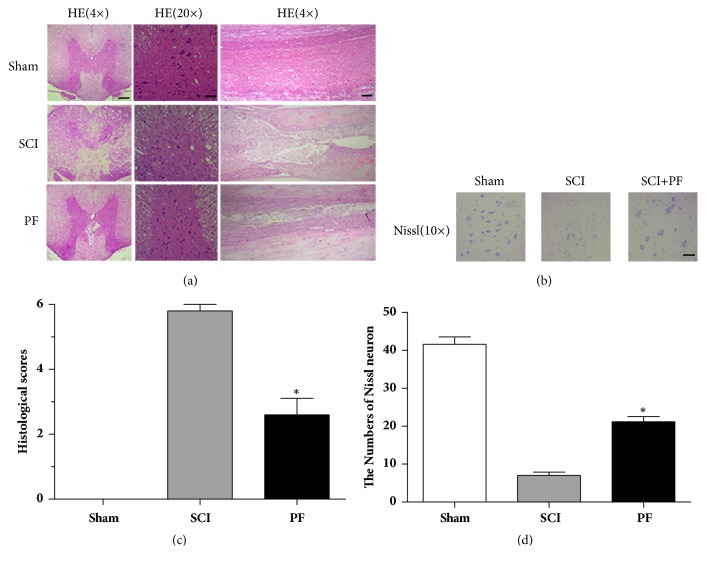
Hematoxylin–Eosin staining (HE) and Nissl staining. (a) Representative section for HE staining at 14 days after injury. Scale bar 200 *μ*m for Magnification 4×, Scale bar 50 *μ*m for Magnification 20×. (b) Nissl staining to display the survival neurons. Scale bar 100 *μ*m. (c) (d) Spine cord injury histological scores and statistics of the Nissl staining results. *∗*P<0.01, *∗∗*P<0.05 compared with SCI group, n=5.

To confirm the neuroprotective effect of PF, spinal cord tissue was HE and Nissl stained. As shown in Figure 2, in injured spinal cord, the center of the lesion was significant and lost its normal structure.

Denaturation, necrosis, and apoptosis were observed in gray and white matter, at transverse and longitudinal sections (Figure 2(a)). Specifically, in ventral neurons, we observed few or no Nissl bodies (Figure 2(b)). Compared to SCI rats, tissues obtained from PF rats had less morphological damage (Figure 2(c)), less areas of necrosis, infiltrated polymorphonuclear leukocytes and macrophages, and increased neuronal survival (Figure 2(d)). The results of HE and Nissl staining suggest that PF treatment reversed the destruction of spine cord.

### 3.3. PF Inhibited Nuclear Expression of NF-*κ*B and Activation of iNOS and COX2 in SCI Rats

**Figure 3:**
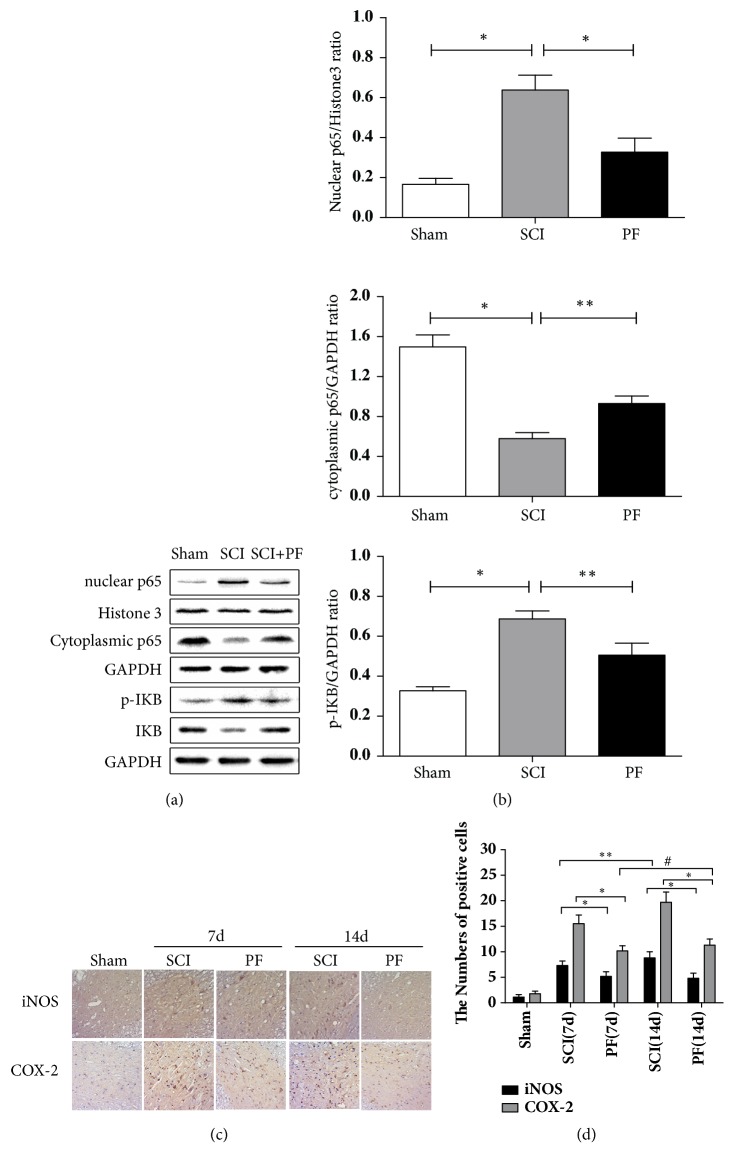
(a) Western blot analysis and quantification data of nuclear p65, cytoplasmic p65, phosphor-I*Κ*B, and I*Κ*B expression in each group of rats. (b) The optical density analysis of nuclear p65, cytoplasmic p65, phosphor-I*Κ*B, and I*Κ*B protein. *∗*P< 0.01, *∗∗*P< 0.05 compared with SCI group, n=5. (c) Immunohistochemistry for iNOS and COX-2 in the Sham, 7d and 14d after spinal cord injury lesion and PF treatment 7d and 14d after injury groups. *∗*P< 0.01, *∗∗*P< 0.05, # P > 0.05, n=5. (d) Analysis of the positive cells.

We performed western blot analysis to examine expression levels of NF-*κ*B and associated proteins. Levels of NF-*κ*B and I*κ*B*α* were detected in the tissue homogenate obtained from rats in all groups. As shown in Figures 3(a) and 3(b), after SCI stress, we determined that nuclear NF-*κ*B was increased, and cytoplasmic NF-*κ*B and I*κ*B*α* (an inhibitory marker of NF-*κ*B) was decreased significantly (P<0.01), compared to Sham rats in spine cord tissue. In contrast, levels of nuclear protein p65 were downregulated after PF treatment (P<0.01), whereas cytoplasmic protein p65 levels were upregulated (P<0.05).

Under normal conditions, without injury, iNOS and COX-2 expression was nearly undetectable. IHC staining indicated that levels of iNOS and COX-2 increased significantly after SCI (Figures 3(c) and 3(d); P<0.01).

These changes were significantly intervened by PF preconditioning. NF-*κ*B-induced inflammation and oxidative stress proteins (iNOS and COX-2)-positive cells were gradually reduced by PF administration 7 d and 14 d after SCI (P<0.01). Additionally, the number of iNOS and COX-2-positive cells in SCI rats was significantly increased with time following SCI (P<0.01 or P<0.05). Next, to further analyze the relationship between the effects of PF and time following SCI, we compared positive cells of 14 d after PF administration to that of 7 d administration. No significant difference was found in iNOS and COX-2 expression after PF treatment at 7 d and 14 d (P>0.05). Taken together, PF may alleviate the oxidative stress in the spinal cord.

### 3.4. PF Alleviated the Nogo-A Activation and Neuronal Apoptosis

**Figure 4:**
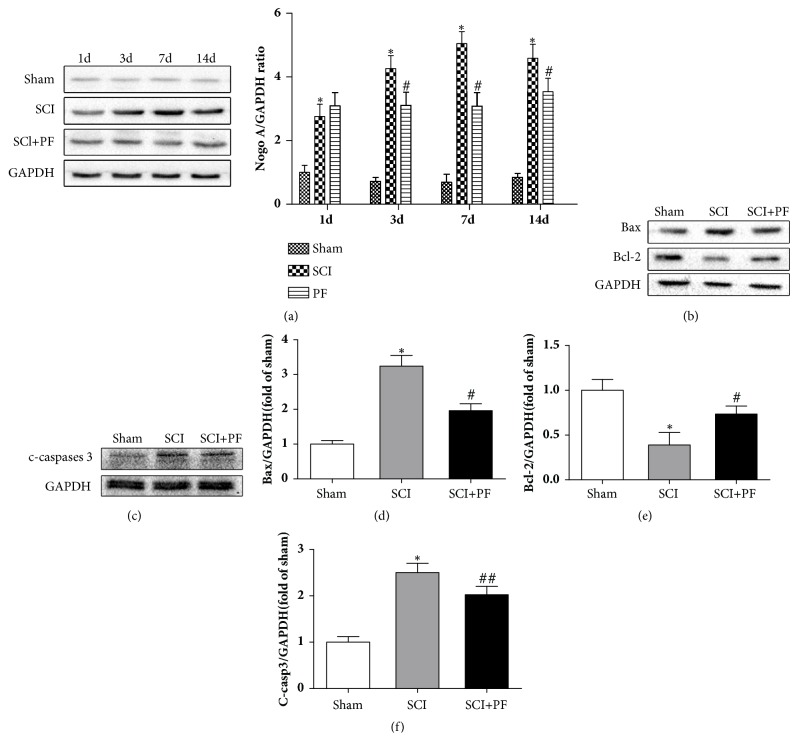
Western blot analysis and quantification data of Nogo-A, Bax, Bcl-2, and c-caspase-3 expression in each group of rats. *∗*P<0.01 versus the Sham group, #P<0.01, ##P<0.05 versus the SCI group at the corresponding time points.

The western blot assay results indicated that the expression of Nogo-A significant increased after SCI and reached the peak at 7 days (Figure 4(a), P<0.05). Meanwhile, we found that PF treatment suppressed the expression of Nogo-A in spinal cord (Figure 4(a), P<0.01). At first day after operation, there was no significant difference in activated Nogo-A expression among SCI, PF groups, and Sham group (Figure 4(a), P>0.05). To further examine the neuronal apoptosis, we determined expression levels of Bax, Bcl-2, and c-caspase-3, three proteins with a crucial role in apoptosis. We found that the proapoptotic protein Bax and c-caspase-3 significant increased, and antiapoptotic protein Bcl-2 decreased after SCI (Figures 4(b) and 4(c), P<0.01). However, administration of PF upregulated the expression of Bcl-2 (P<0.01) and downregulated the expression of Bax (P<0.01) and cleaved-caspase 3 (P<0.05).

### 3.5. PF Protected PC12 Cells from *H*_2_*O*_2_-Induced Injury and Suppressed Inflammatory Responses

**Figure 5:**
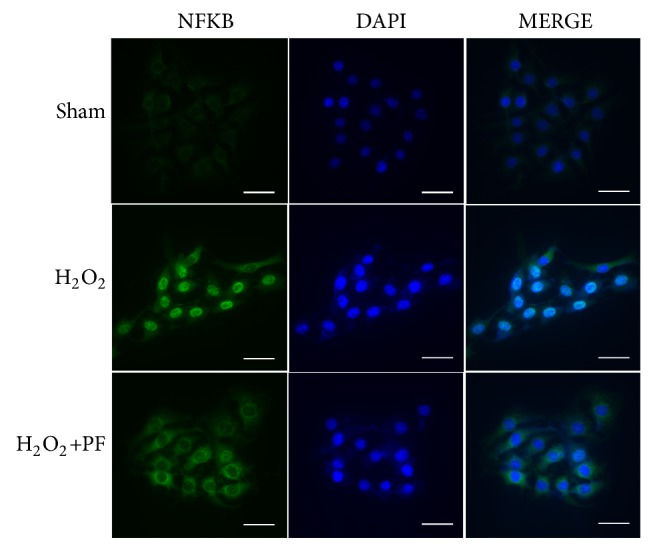
Immunofluorescence staining results of NF-*κ*B for PC12 cells. NF-*κ*B transcription is observed by (green) fluorescence localization; the nuclear is labeled by DAPI (blue). The bright blue nuclear in the right column is considered as positive staining NF-*κ*B which is activated; magnification was ×20.

**Figure 6:**
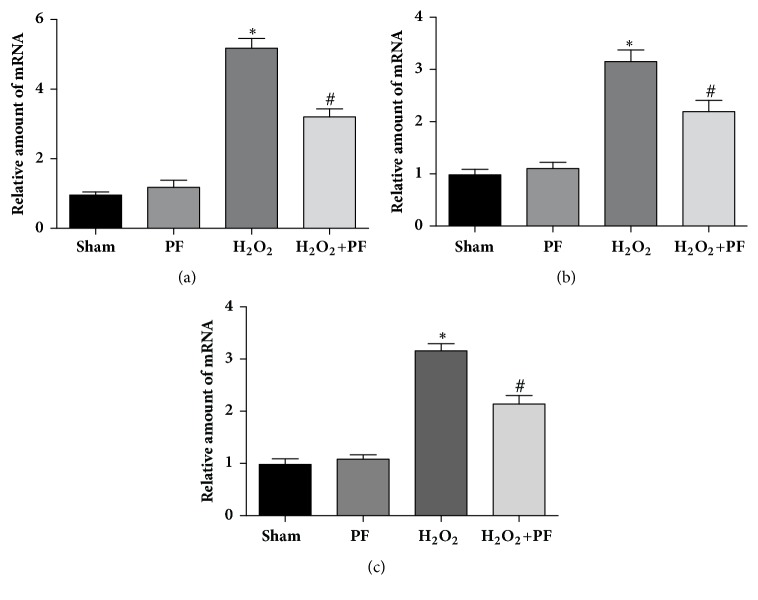
PF reduced H_2_O_2_-induced inflammatory factor in PC12 cells. PF prevented the increase of the mRNA expression of the inflammatory genes, such as IL-1*β*, IL-6, and TNF-*α*. PC12 were pretreated with PF (200 *μ*M) for 2 h and then cultured with 200 *μ*M H_2_O_2_ for 2 h. *∗*P<0.01 versus the Sham group, #P<0.01 versus the H_2_O_2_ group.

To further reinvestigate the relevance of* in vitro *findings and* in vivo *systems, we examined the effect of PF in oxidative-injured (H_2_O_2_) PC12 cells. Results from green fluorescence protein orientation showed NF-*κ*B aggregation in the cell nucleus after H_2_O_2_ stimulation, indicating the nuclear translocation process of NF-*κ*B (Figure 5). Next, we have detected levels of inflammatory factors (TNF-*α*, IL-6, and IL-1*β*) with RT-qPCR. Compared to unstimulated cells, stressed PC12 cells had higher mRNA expression of proinflammatory cytokines including TNF-*α*, IL-6, and IL-1*β* (Figure 6, P<0.01).

Upon pretreatment with PF, upregulation of TNF-*α*, IL-6, and IL-1*β* at mRNA levels was prevented (Figure 6, P<0.01). These results show that PF may suppress neuroinflammatory responses via preventing nuclear translocation of NF-*κ*B and reducing induction of proinflammatory cytokines.

### 3.6. PF Protected the PC12 Cells from *H*_2_*O*_2_-Induced Injury and Increased PC12 Cells Viability

**Figure 7:**
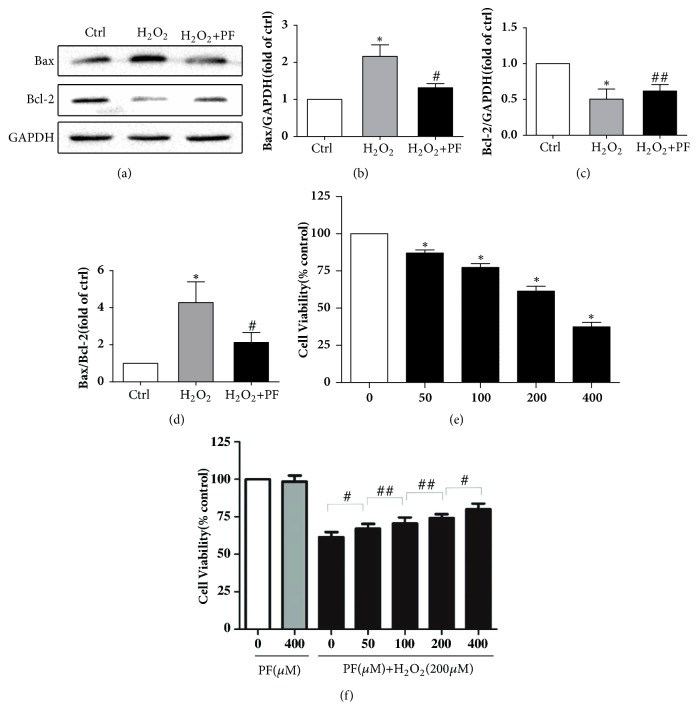
PF neuroprotection upon oxidative stress in PC12 cells. PC12 cells were either treated with H_2_O_2_ at indicated concentrations for 2 h. PC12 cells were treated with the indicated doses of paeoniflorin in the presence or absence of 200 *μ*M H_2_O_2_. (a-d) Western blots to detect cell apoptotic. *∗* P< 0.01 versus the control group, #P< 0.01, ##P< 0.05 versus the H_2_O_2_ group, n=5. And (e, f) Cell Counting Kit-8 analysis to detect cell viabilities. *∗*P< 0.01 versus the control group, #P< 0.01, ##P< 0.05 versus other treatment groups, n=5.

In consistent with the result of western blot* in vitro*, PF preintervention significantly reduced H_2_O_2_-induced Bax levels and maintained Bcl-2 levels, which may be related to increased cell viability. Cell viability was determined using Cell Counting Kit-8. Predictably, PC12 cell viability decreased to different degrees after H_2_O_2_ stimulation at concentrations of 0, 50, 100, 200, and 400 *μ*M for various intervals (Figure 7(e)). Next we examined the cytotoxicity of PF and found no significant difference in cell viability between controls and the single PF treatment group (400 *μ*M) (P>0.05). To further confirm the neuroprotective effects of PF on oxidative stress, we pretreated PC12 cells with PF (0, 50, 100, and 200 *μ*g/ml) 2 h before H_2_O_2_ stimulation (200 *μ*M) for 2 h. We found that PF markedly inhibited H_2_O_2_-induced oxidative stress in a concentration-dependent manner (Figure 7(f)), suggesting that PF has a cytoprotective effect.

## 4. Discussion

Acute SCI is usually caused by injuries from traffic accidents and falling accidents and can result in permanent disability and serious injury. It is well known that motor function recovery following SCI is difficult. Operative treatment has been shown to be effective in relieving symptoms of nerve compression. However, there is still a dispute regarding effective methods to hinder secondary injury reactions and protect spinal neurons from trauma.

PF is the primary active chemical extracted from the roots of Paeonia plants and has been considered to exert protection effects on the central nervous system [21, 26]. Moreover, PF was reported to improve the behavioral performance and memory in the 1-methyl-4-phenyl-1, 2, 3, 6-tetrahydropyridine- (MPTP-) treatment mouse model of Parkinson's disease and alleviate cerebral ischemia injury [27].

PF has been shown to pass through the blood–brain barrier rapidly and protect the central nervous system by inhibiting NF-*κ*B in glial cells [28]. In this study, we used high purity (99%) PF to ensure we observed its full effects and reduced errors. Our primary aim was to verify that PF treatment could reduce changes in inflammation-related proteins after SCI and, thus, protect the neuron from injury.

Inflammatory response and oxidative stress damage are important mechanisms of neuronal injury after SCI. Additionally, it is well known that NF-*κ*B (p65) and NO play a pivotal role in secondary injury after SCI. Under normal conditions, NF-*κ*B is restricted in the cytoplasm by the inhibitory protein I*κ*B. A previous report indicated that ischemia induced overexpression of NF-*κ*B and degradation of its endogenous inhibitor I*κ*B*α* [29]. Ample evidence suggests that PF can suppress 6-hydroxydopamine- (OHDA-) induced NF-*κ*B translocation and mitochondria-mediated apoptosis of PC12 cells [30]. Our results are consistent with those of previous studies. Specifically, after SCI, we observed overexpression of nuclear p65 (NF-*κ*B) and decreased levels of cytoplasmic p65 and its endogenous inhibitor phosphorylated I*κ*B*α*, and these changes were not observed in rats pretreated with PF. We observed strong activation of NF-*κ*B nuclear translocation following H_2_O_2_ stimulation, which was inhibited by PF treatment, suggesting that the NF-*κ*B signaling pathway plays an important role in PF-mediated neuroprotective effects.

COX-derived oxidative stress and NOS-derived nitrogen reactive species can be major factors in age-related inflammation [31]. Additionally, it has been hypothesized that an excess of NO, generated by iNOS, exposes gray matter neurons to highly toxic chemicals [32, 33]. COX-2 is associated with proinflammatory stimuli, such as TNF-*α*, IL-6, and IL-1*β* [34]. Additionally, NOS activity is modulated in early stages after NF-*κ*B activation [35]. Furthermore, studies show that knockdown of iNOS in rat with SCI results in enhanced motor function compared to that in controls [32]. Our immunohistochemistry results demonstrate that PF treatment downregulated iNOS and COX-2 expression. However, the relationship of time of PF treatment is still unclear. In a previous study, we demonstrated that PF could modulate Th1/Th2 cell imbalances by regulating the expression of the transcription factors T-bet and Gata-3 in intervertebral disc degeneration rats. IFN-*γ* and TNF-*α* of PF groups were significantly decreased compared to those of the model group in a time-dependent manner over 3, 7, and 21 days. We previously found that PF could inhibit nucleus pulposus cell apoptosis and inhibit caspase-3 and caspase-9 activation by regulating Bcl-2 family protein [15]. To confirm this* in vitro*, H_2_O_2_ was used to simulate oxidative stress damage to activate NF-*κ*B signaling pathways in PC12 cells. Additionally, in this study, we used RT-qPCR to further demonstrate the anti-inflammatory effect of PF on PC12 cells and found that upregulation of TNF-*α*, IL-6, and IL-1*β* was prevented at the mRNA level. In our* in vitro* experiment, PF treatment downregulated the expression of Bax and upregulated the expression of Bcl-2. These results are all in agreement with those of Wu and Jin, who reported that PF treatment can inhibit apoptosis of NPC cells mediated by H_2_O_2_ [36]. The results of CCK-8 further confirmed that PF plays a cell protective effect in neuroinflammation damage.

When SCI happened, we observed that spinal structure was destroyed with great loss of nerve fibers. As one of the myelin related proteins in oligodendrocytes, Nogo-A is believed to be an inhibitor of nerve fiber impair when axon is injured [12]. It is generally accepted that C-terminal region (Nogo-66) of Nogo-A transduces the inhibitory signal into the cell interior of neurons by combining with Nogo-66 receptor (NgR). The research reports that treatment with Nogo shRNAs, which can knockdown Nogo gene expression, improved function recovery of spinal cord injury rats [37]. In the present study, we found that PF treatment suppressed Nogo-A expression in the injured spinal cord. Meanwhile, we observed that tissues obtained from rats treated with PF had increased neuronal survival in the ventral horn of the spinal cord compared to SCI rats. This demonstrates a significant protective effect with less area of necrosis, consistent with the report showing that the neurological deficits and a robust loss caused by middle cerebral artery occlusion could also be inhibited by PF pretreatment [22]. After 1 week, treatment with PF progressively increased the locomotor function compared to SCI rats without PF treatment.

However, the present study still has some limitations. We focused on motor function recovery, which is dependent on motor neurons in the ventral horn. In paeoniflorin group, we found recovery of spontaneous voiding in some rats, but we did not evaluate the bladder function or calculate residual urine. We may add this research in future study. Other studies have described enhanced cell viability results after PF treatment compared to that observed in our study [30]. This may be due to the purity of PF, cell subculture, or the degree of H_2_O_2_ damage. Future research will be done on PF combined with nerve growth factor with epidural administration and synthesis biomaterials of sustained-release hydrogel. Additionally, we will attempt to determine the upstream and downstream sequence concrete mechanism of NF-*κ*B signaling pathway with PF intervention.

Although the neuroprotective effects of PF have been reported in animal models of neuropathology, no systematic investigation has reported that PF improves the motor function of SCI. We suggest that PF improves functional recovery of SCI rats for the first time. Currently, the major drug treatment for SCI is methylprednisolone (MP) aggressive therapy, which reduces inflammation response and neural edema of the spinal cord [38]. However, hormones cause many side effects, such as electrolyte disorder, aseptic bone necrosis, osteoporosis, and digestive tract symptoms [38]. We believe that PF has potential as a naturally derived treatment to reduce the progression of secondary injury and could be used as a therapeutic strategy in SCI in the future due to its dual effects of anti-inflammatory and neuroprotection.

## 5. Conclusion

Our study shows that treatment with PF reduced neuron axon trauma and histopathological damage of the spinal cord and increased the survival of neurons in the spinal cord lesions. Inhibition of NF-*κ*B signaling pathway may be involved in the neuroprotective effect of PF which enhanced the functional recovery of hind limbs of acute SCI model rats. PF treatment may be a therapeutic strategy in spinal cord injury. However, its detailed mechanism needs further study.

## Figures and Tables

**Table 1:** Primer sequences for real-time PCR.

Name	Primer	Sequence
IL-1*β*	Forward	GACAAGAGCTTCAGGAAGGCAGTG
	Reverse	CACACTAGCAGGTCGTCATCATCC
TNF-*α*	Forward	TCCAGAACTCCAGGCGGTGTG
	Reverse	GTTCAGTAGACAGAAGAGCGTGGTG
IL-6	Forward	AGCCACTGCCTTCCCTAC
	Reverse	TTGCCATTGCACAACTCTT

## Data Availability

The data used to support the findings of this study are available from the corresponding author upon request.
